# An Essay upon the Relation of Bilious and Yellow Fever

**Published:** 1856-10

**Authors:** Richard D. Arnold

**Affiliations:** Professor of the Theory and Practice of Medicine in the Savannah Medical College


### SELECTIONS.

*An Essay upon the Relation of Bilious and Yellow Fever—prepared at the request of, and read before, the Medical Society of the State of Georgia, at its session held at Macon on the 9th April,* 1856. By RICHARD D. ARNOLD, M. D., Professor of the Theory and Practice of Medicine in the Savannah Medical College.

The subject about to be discussed by me, in compliance with the appointment of the Society, is—THE RELATION OF BILIOUS AND YELLOW FEVER.

At first sight, this may appear to be a question of very little practical utility, and one upon which there is little contrariety of opinion. But when I recollect the change which years and experience have wrought in my own opinions, and when I see taught in medical text-books views so totally dissimilar to those I now entertain on the subject, I have not been unwilling to endeavor to convey to the society what I conceive to be the true relations between Bilious and Yellow fever. In doing so, I shall not pretend to give a history of either Bilious or Yellow fever, nor shall I bring any bibliographical array to support my views: not that I underrate the value of books, for without a proper knowledge of what has been learned and taught before our day, we would be little better than blind mill-horses, constantly pursuing one narrow circuit; but, because the subject is a strictly practical one, which must be decided by the weight of testimony.

Each observer must bring in his mite of observation, add his grain to the mound of true knowledge; "and, as the laborious ant—

*———Trahit quodcunque potest, atque addit acervo*
*Quem struit, haud ignara ac non incauta futuri.*"

While Yellow fever has never been known to prevail in climates and localities where Bilious fever was not endemic—Bilious fever, in its most malignant form, is known to prevail where Yellow fever has never been seen. Having practiced for more than twenty-five years in one of those localities where Bilious fever is annually endemic, and Yellow fever only an occasional visitor, I feel that I have been placed in a position which has given me some advantages in treating of this subject. Two opinions have prevailed amongst those who have judged from this fact: first, that Yellow fever, occurring as it does where Bilious fever is endemic, is but a higher grade of the same disease, produced by the same causes, acting in greater intensity; second, that Yellow fever is a disease *sui generis*, having no analogy nor connection with Bilious fever, not produced by any local causes, but *invariably* imported from abroad, therefore to be kept away by quarantines and all their inhuman vexations and costly consequences. I shall proceed to consider these two opinions in their order.

1st. *Is Yellow fever only a higher grade of Bilious fever?*—The first time I ever saw a case of Yellow fever was, while a pupil of the late Dr. Wm. R. Waring, in the summer of 1827. No man in the southern country was better acquainted with our fevers than he was. He had seen them in various localities while a surgeon in the army; he had seen them in all their violence in our own city—before the dry culture system, by removing the culture of rice, with its concomitant evils, from under our very door sills, had so favorably modified the type of Bilious fever as met with in the city proper; he had been through our then recent epidemic of Yellow fever of 1820, from the beginning to the end; in company with the distinguished Chervin, he had conducted a series of post-mortem examinations of Yellow fever subjects, and he could thus "speak by the card." During the fall of 1827, Yellow fever broke out in our city: it did not prevail very extensively; for, occurring late in the season, its mighty destroyer, frost, put an end to it before it had time to spread extensively.

It was my privilege to conduct all the post-mortem examinations made by my preceptor during that season. Is it any wonder that I should have considered him my medical Gamaliel, and have sat reverently at his footstool? Among the opinions held by my distinguished preceptor was this identical one, that Yellow fever was but a higher grade of Bilious fever. *He* had imbibed this from *his* preceptor, the celebrated Rush; it descended in a straight line to me, and many years rolled by before I dared to question its accuracy; and I did not do so, until repeated observations had given me data on which to base my belief. Such is the power of authority, which too often trammels us in our researches. In fact, in our views of general affairs in this world, of politics, of religion, &c., there are very few who can truly apply as a motto—

"*Nulius addictus jurare tu verba magistri.*"

If Yellow fever were only a higher grade of Bilious fever, we ought to see it "cropping out," whenever there was any unusual intensity, or any greater prevalence of the latter.

I have witnessed every epidemic in the city of Savannah, from the year 1830 up to the present time; I have often known and seen Bilious fever of a malignant congestive type; for fifteen consecutive summers I was the attending physician of the city hospital, whither the worst cases of our ordinary climate fever are conveyed.

When year after year, I met with malignant and fatal cases of Bilious fever, and yet with not a single one of Yellow fever, I began to doubt whether or not I was right in my opinion. Occasionally a few cases of Yellow fever would occur, at intervals of years; these I studied with intense interest. From 1830 up to 1839, I never saw a case of Yellow fever in the city. Its characters were indelibly imprinted on my memory from the experience 1827. In 1839, the city of Augusta was ravaged by this scourge: it was denied at the time that Yellow fever prevailed there. In the last of August, a patient, from Augusta, entered the city hospital, and died in a couple of days. My then colleague, Dr. P. M. Kollock, and myself examined the body, and found the unmistakable post-mortem appearances of genuine Yellow fever. A short time afterwards, a patient from Charleston entered, and died, and after death presented the same appearances. It is worthy of recollection, that although these cases were placed in the wards of a hospital filled with Bilious fever patients, there was no propagation of the disease. I still look back upon the year 1839, as the sickliest season I have ever experienced in Savannah, with the exception of our terrible epidemic of 1854. Old inhabitants will recollect it as the driest summer on record, when turnips were planted in the bed of the Savannah river opposite Augusta. It was also a hot summer. Bilious fever prevailed over the whole country, and in a malignant form. Contrary to what would seem the fact at first view, such a season was peculiarly calculated to generate the malaria which is the generally acknowledged cause of Bilious fever. It is conceded that mere moisture will not produce malaria; but mix vegetable matter with water, and subject it to heat, and the most malignant malaria will be generated. That year, swamps and ponds which had been covered with water since they had been known to the white man, were dried up, and the vegetable *debris* which had been precipitating to their bottom for years and years, were exposed to the action of the sun and air, and consequently were decomposed, and generated malaria. Now, Bilious fever prevailed with great violence in our city from early in July. I cannot imagine more favorable circumstances for the spread of Yellow fever than accompanied the introduction of these two cases in our city. Later in the season, I did meet with several cases of Yellow fever, but they were so few in number that I did not consider them as entitled to be considered epidemic. They were isolated, occurred in different parts of the city, and had not the slightest connection with the cases of the hospital. I considered them sporadic, and they most undoubtedly originated on the spot.

I met with one solitary case of Yellow fever, with black vomit, in the fall of 1840. She was an unacclimated foreign lady, who had not stirred out of the city during the whole summer, nor had she even peeled a banana from Havana.

In March, 1841, a case was brought to the hospital from Demerara, and in October of the same year a case occurred in my private practice, both of which were reported by me in the American Journal of the Medical Sciences for October, 1842.

A few cases occurred at the hospital late in the fall of that year. I found the post-mortem appearances so similar in all the cases I had examined, from 1827 up to this time, that I was convinced that Yellow fever must be a disease *sui generis.* It was with increased interest, that during the summers of 1843, 4, 5, 6, 7, 8, 9, 1 examined every case of fever which died at the hospital. Neither during life, while attending them, nor after death, did I find any signs to make me ever suspect that Yellow fever had existed. With the exception of a sporadic case in June, 1852, I met with no Yellow fever until the fall of 1852. Late in September, an unacclimated painter was attacked with it in the northeastern portion of the city: he had been working here all summer, and had no connection with Charleston or Havana. He was removed to the hospital after he had thrown up black vomit, and he died. I had resigned my post as physician there in 1850, and was not attending. An autopsy was made, at which I was present. Before it was done, I stated to the attendants what morbid appearances I expected to see; and they turned out exactly as described beforehand. The fever began to show itself in several places about the middle of October; but, fortunately, a frost early in November cut it short. I examined several subjects who died of it, and found the same peculiar morbid appearances. In 1854, it was my lot again, as in 1852, to have the first Yellow fever patient. I was called in on the night of the 3d August, and he died on the morning of the 5th, after having discharged quarts of genuine black vomit. My last case of Yellow fever, with black vomit, died on the 27th November. In the intermediate time, I had seen hundreds of cases of genuine Yellow fever.

I had made post-mortem examinations in the beginning, the middle, and the end of our epidemic, under the broiling sun of August, the more temperate atmosphere of the latter end of September and October, and the almost cool temperature of November, and I found nothing new. From the beginning to the end, I found the same morbid appearances. Of course I do not mean that each case was an exact copy of the other; but just as all cases of genuine Typhoid fever present the same morbid appearances, although the patches of Peyer may be more ulcerated in one case than in the other, or in some cases they may be enlarged without being ulcerated.

Now, the morbid appearances after Bilious fever have never, in my experience, approximated those after Yellow fever; and the symptoms during life have presented wide and marked differences. Let us devote a little attention to these two conditions.

It is well known that all fevers have many symptoms in common in their beginning, such as headache, lassitude, pain in the limbs, &c.; and that merely from such symptoms it would be impossible for the most experienced and skilful practitioner to diagnosticate the particular kind of fever presented to him. He must wait the progress of the case, and the development of the characteristic symptoms, before he can decide. Of course I speak of the inception of the disease. Certain fevers come on, as a general rule, more suddenly than others; but the rule is not invariable, and we would be at a loss to make a correct decision if we depended merely on the first phenomena of febrile disturbance.

Moreover, some cases of well-known specific exanthematous diseases are developed so imperfectly that we are at a loss to decide, positively, whether or not the patient has had the genuine disease. Every practitioner of any experience must have met with such cases of Scarlet fever and Measles. Yet no one has ever, in latter days, denied that they are distinct and peculiar diseases, although a little more than a century ago Measles was confounded with Small-pox.

I do not deny that, when no suspicion is aroused, sometimes the first notice the physician has that he is treating a case of Yellow fever, is the appearance of the fatal black vomit. But even in epidemics of Yellow fever, black vomit often supervenes when the patient has apparently passed the point of danger and offered no untoward symptoms.

Nor must it be supposed that all cases of genuine Yellow fever appeared in one stereotyped edition. There was every variety of grade and intensity, from the ephemeral attack of twelve hours of fever, followed by speedy convalescence, to the more prolonged paroxysm of seventy-two hours, ushering in a malignant or a fatal case. Yellow fever is essentially a fever of one paroxysm; but that paroxysm is of very unequal duration, as just intimated. Now, if the access of fever should not be very marked, it could not be distinguished at first. Again: there are some cases which are ushered in with such marked symptoms that your suspicions would be at once aroused. The first case with which I met, in 1854, was one of this nature. There had been no unusual severity in the fevers which had occurred up to that time. The summer had been the very hottest I had ever experienced, and what is very rare many fatal cases of *coup de soleil* had occurred. I was called to see my patient at night (3d of August); he had taken comp. blue pill: he offered the usual symptoms of fever—pain in the head, in the loins, over the upper part of the sacrum, down the thighs; a hot, dry skin, and accelerated pulse. I directed a demulcent drink, and that a dose of castor oil should be administered the next morning early. On my visit the next morning, I found him with a raging fever; intense headache; blood-shot, shining, watery, smoke-affected eyes; a full, bounding, but not very frequent pulse; a constant retching, and quite delirious. The landlady said to me, "Doctor, what kind of a fever is this?" I replied, "It is first-cousin to Yellow fever." I bled him, and applied a blister to the epigastrium, and directed cold demulcent drinks. The fever continued unabated all that day and the ensuing night. On the morning of the 5th of August, on my visit, they showed me a large wash-hand basin filled with matter, which I pronounced black vomit. He continued to eject large quantities of it, and at noon he died. This man was a carpenter by trade, a northerner; it was his first summer south; he had been working on the roof of house which was just finishing, and before he had moved to the place where he was then boarding, had lived in Curry town, the extreme south-western portion of the city, and had walked nearly a mile two or three times daily to and from his work, which was in the north-eastern portion of the city, through the broiling sun. It is again to be noticed that not a single other death occurred in that house during the season. After the epidemic became a fixed fact, and cases had occurred all over that section of the city, two of the inmates had the fever, but it was in a mild form. There was not any loop-hole whereon to hang even a suspicion that this man's disease had been contracted any where out of the limits of the city—no "low, long, black-hulled schooner" had just arrived from the West Indies to afford an easy solution of how Yellow fever had attacked a denizen of Savannah. The house was a mechanics' boarding-house. A great panic ensued; but I am yet to learn that any boarder contracted the disease from this case.

The next afternoon, the 6th August, I was called by Dr. Jas B. Read to see a case on the extreme eastern edge of the city, many squares distant from the first case. The patient was a young German girl, entirely unacclimated; the house where she lay was on the eastern bluff of the city, overlooking the low swampy grounds in that direction; it was amply ventilated, standing isolated in a large lot. She had been engaged sewing decorations at the theatre, distant about three-quarters of a mile, and had walked to and from it in the hot sun, during the whole season. Unfortunately there was no doubt about the case; she was moribund, with black vomit thrown up all about her.

On the afternoon of the 5th, I was called to see a patient in Congress street, about a hundred yards to the north-east of my first patient, but in a different street. His fever did not attract my attention particularly. He was of a lymphatic temperament, a northerner who had resided several years in the city, and whom I had attended some summers before in a very severe attack of bilious fever. His fever was not very high; he complained of pain and languor. I gave him a dose of blue pill, and directed oil the ensuing morning. On the 6th his medicine purged him and he was better. On the 7th, when I called to see him, he had left the house and gone to his business. On the evening of the 8th I was called to him ; I found him with a slow pulse, a cool skin, and a constant retching, ejecting glairy matter, and no bile.

For the first time, I suspected Yellow fever, and that the cessation of the fever had been the calm which follows the single paroxysm of that fever. I ordered a blister to epigastrium, ice to suck, and iced gum water for a drink, and my alterative powders, (two grains of calomel, and one sixth of a grain of opium,) every two hours. On the 9th, he was much the same, except that the prostration was greater, so as to seem to threaten death from sheer exhaustion. Towards night, I discovered flocculi of black vomit in his vomit. He continued to throw up the black vomit mixed with a good deal of mucus, all that night, and all the next day, and died on the night of the eleventh. The quantity ejected was not very great, and it was thrown up with a great deal of straining and mixed with mucus. He sank away gradually and gently, like one yielding to the effects of a depressing poison, without the power of reaction.

Let us contrast the first case and this. The first case occurred in an unacclimated subject—it was violent from beginning to end. The last occurred in an acclimated subject—it was slow in its progress, less marked in the first stage, but running its stage of calm and secondary fever as is most generally seen where death does not occur during or just after the paroxysm of the fever.

Now, no *fact* is more notorious than that acclimation to a warm latitude diminishes the susceptibility to yellow fever, and that it is far milder in those who have constantly resided south, summer and winter, than in those who have not; and who are consequently unacclimated. By this time, the eleventh of August, I had been called to many cases, all in the north-eastern part of the city, but in separate houses and different streets —not in any ways connected with each other, and I could not doubt that we had a different fever to contend with than a bilious remittent fever, and I will now proceed to state the symptoms which brought me to that conclusion. Of course I will state what was the general type.

The invasion of fever was more sudden than in ordinary Bilious fever, and although all fevers will be found to have a cold stage of some kind, it was not well marked in these. There was intense pain in the back, over the last lumbar vertebra and upper part of the sacraum and extending down the thighs along the sciatic nerve. The pain over the eyes, in the frontal region, was excruciating ; the eyes were watery, shining, sometimes injected, sometimes not, with the upper lid partially drooping, like one whose eyes were watering from a quantity of smoke. The skin was intensely hot and very dry ; the stomach was very irritable ; the ejecta were either a serous fluid, bluish green, as if blue vitriol had been dissolved in water ; or a glairy, viscid, tenacious mucus. But the pulse was not disturbed in accordance with the general perturbation, it seldom being over a hundred beats to the minute, and very often not more than eighty or ninety. This symptom I have been disposed to look upon as very characteristic. This febrile state lasted from twelve to seventy-two hours, on an average of about thirity-six hours, and was succeeded by a cessation of these symptoms, and an apyrexia, but without any critical evacuation whatsoever. After this, in fatal cases, black vomit came on immediately, or it was ushered in by increased irritability of the stomach, it becoming intolerant of the mildest ingesta, by a constant empty straining, and by the most acute sensibility of the epigastrium to any pressure. With this, in most cases, there was the most remarkable depression of strength ; in some cases, several hours of a most perfect calm succeeded the paroxysm, and there was nothing to rouse suspicion of danger but a slow pulse, it generally sinking to forty or sixty beats in the minute. When black vomit supervened, a few hours terminated the case. With all this manifest affection of the stomach, the brain, as a general rule, did not sympathise. The intellect was not affected, until the last closing scenes of life, when the brain gave away in common with the rest of the organism.

This, considering the violence of the febrile paroxysm, must be considered one of the characteristics of Yellow fever. The thrist was great, but the tongue was not generally parched. At this period of the epidemic, the fatal cases terminated quickly ; black vomit came on within three r four days, and the patient seldom survived beyond the fith day. When e did so, the chances were greatly in his favor.

I have sketched only the prominent symptoms. Do they differ from those presented in a case of Bilious fever ? Let us take a well developed, well marked case, with which to make our comparison.

A Bilious fever is almost always ushered in with a pretty distinctly marked chill. There are certain symptoms, as stated before, which are common to the various forms of fever, such as weariness, head ache, &c.; but the invasion of these pains is not so sudden in a bilious fever. After the cold stage, there is an evident reaction of the system, and a hot stage ensues. This, again, is followed by a third stage, viz : one of sweating ; the fever then abates, with a distinct critical evacuation, either by urine or by perspiration ; and in a few hours it again begins to increase, and having attained its height a sweating stage again follows ; and so the fever goes on, the remissions becoming shorter, the stages less marked, until the system sinks under it ; or the paroxysms become lighter, the remissions so marked and distinct that they slide into intermissions, and the patient recovers.

MARKED PERIODICITY is the distinctive characteristic of Bilious fever, as far as it has come under my observation.

But there are certain symptoms attending the paroxysms which are quite distinctive. The pulse becomes more accelerated in Bilious fever, ranging far above one hundred, increasing as the fever increases, becoming slower as it abates, presenting, as verified by me, in scores of cases, a variation of forty beats to the minute between my morning and evening visit.

There is the headache in Bilious fever, but is not of that intense supra-orbital character as in Yellow fever, and is more diffused over the anterior portions of the brain. There is very frequently great irritability of the stomach in Bilious fevers, and the stomach ejects great quantities of bile. Sometimes the bile may assume a greenish color. Very often, a severe attack of Bilious fever may present its paroxysms so marked, and the remissions so distinct, that you could with propriety class it in the intermittent variety ; still the three stages, of cold, heat, and sweating, can be easily traced and marked in the form of Bilious fever. Now, if Yellow fever was but the highest grade of Bilious or Climate fever, we ought to find the worst cases of the latter closely approximating, if not running into the former. But what are the facts ? The congestive type of Bilious fever (as witnessed by me in hundreds of cases) is unquestionably the very worst type of that fever. Every year, however healthy, affords cases of it in those individulas who have been exposed to swamp miasma in their avocations. Watchmen are required at the wharves under the bluff of the city. Savannah lies on a high bluff, forty feet above the level of the tide, and fronts to the north. Northward is a low alluvium extending in a direct line due north, for fully four miles before the high ground of South Carolina is reached. Hutchinson's Island, immediately opposite to, and north of the city, is under the dry culture contract, which prohibits the planting of rice within a mile of the Exchange; but beyond the back river and on the Carolina side, are vast bodies of land, fully from two to four miles through in a northward direction, and extending east and west for about twelve miles, which are cultivated in rice with all its concomitant moisture. To the northeast of the city, these lands extend to the limit where the water becomes brackish and unfit for the culture of rice. To the direct east of the city, and beyond the limits of the dry culture contract, and on the Georgia mainland are many hundreds of acres of land cultivated in rice. To the north-west of the city, the alluvium takes a bend to the north, affording in that quarter some of the finest rice plantations in South Carolina. What constitutes the defence of Savannah against the malaria of these low grounds? I answer; that, fortunately, almost the whole northern front of the city is defended by a high row of brick storehouses rising some twenty or thirty feet above the level of the plain on which Savannah stands; which storehouses are not inhabited, and thus afford a *material* bulwark against the introduction of malaria into the city. To the north-east of the city, this protection is not afforded, because the storehouses have not been built up in compact mass *above* the level of the plain of the city, as they have been at the portion of the front more westwardly. In this portion of the city there are many dwelling houses on Bay-street which are not protected; whenever the winds prevail from the north or north-east, those houses have invariably, and I speak advisedly from many years' experience, afforded the first cases of Bilious fever, and the most malignant types of it every year. I have a patient who lives in this locality. About six years since he moved into his house, and he and his whole family (a wife and three children,) were desperately ill of Bilious fever. I advised him, nay, insisted upon it, giving my reasons, that in the sickly season, when malaria was generated, (say from the first of June until a frost in the fall,) he should keep all the windows on the north side of his house closely shut by the sashes, from early in the evening until the sun was high up in the morning. He has *done* so, to the exemption of his family from fever, and the great curtailment of my professional fees. Such has been my advice to all persons inhabiting houses exposed in a similar manner, and I distinctly aver, that where the advice has been followed, the same result has obtained.

Those individuals whose *liberty* is the practical one so much sighed after by the pseudo-philanthropists of the North, of working or starving, are the ones who take the perilous occupation of watching at night under the bluff, and who are thus exposed to the malaria which may be blown from the north-east, the north, or the north-west, just as the wind may set.

The summer of 1855, was the healthiest I have ever known in the city. Fevers did not rise above the grade of intermittent, as a general rule, yet I met with two cases of congestive fever, both of which were fatal within four days from my first visit, and each individual had contracted his fever, from exposure at night and early in the morning, in the very locality I have pointed out. They were the fac simile of cases occurring more or less frequently every year; they were malignant, and they were fatal; but they offered not the slightest resemblance to Yellow fever. The fever was high, the pulse was accelerated up to 120 to 140 in the minute; while the skin was hot to the touch, it was covered at times with moisture, standing out in great beads of sweat. The brain was affected with stupor from the very commencement. When the fever remitted, which it did in the morning, and notably on the morning of the alternate day, the brain would become relieved in a measure, but as the fever exacerbated it would again become oppressed. These cases terminated in a stupor many hours before death. Perspiration in the very heighth of the fever, I consider as a very common symptom of the congestive form of Bilious fever, and I always consider those cases most dangerous which show this symptom, while there are stupor, an accelerated pulse, and an intensely hot skin. Whilst the cases which terminated favorably have the paroxysm of fever resolved by a critical sweat, with an abatement or cessation of the other febrile symptoms; I have time and again seen a man *in articulo mortis*, with a pulse so accelerated that it could not well be timed; in a profound stupor, and with the sweat standing out on his skin in great drops; and this condition of affairs had not supervened just before the patient became in *extremis*, but had gradually come on in the last exacerbation of fever. There are other cases which come under the category of pernicious or malignant intermittents, or congestive chills. I have known a patient in a state of perfect apyrexia in the morning, to die in the afternoon. These are the most malignant forms of climate fever met with in this city. I may fail to convey an idea of their real character, but it is from want of power in my pen, not from want of their total dissimilarity to Yellow fever.

Late last fall, but before a frost, a watchman on the Charleston wharf, at the northwestern portion of the city, was found early in the morning lying in a state of complete insensibility. The night had been a stormy one; he had had intermittent fever, but had persisted in going to his work in spite of the remonstrances of his wife. He was carried to his house on the brow of the bluff, and I was sent for. I found him in a complete stupor, with his pulse nearly gone, his extremities icy cold, his whole periphery cool, skin mottled, purplish, or rather in some parts bluish, with a clammy sweat, hurried respiration, and in short, in what I considered a dying condition. I had him stripped and rubbed dry, had dry heat applied to the surface, sinapisms to every available point, and a large blister to the epigastrium and one to each leg. Friction was applied continuously for some hours; after awhile he was enabled to swallow: I gave him hot brandy toddy every half hour, and calomel two grains, and opium one-sixth every two hours. Reaction gradually took place, and the next day, about twenty-four hours after he had been brought home, he spoke. From that time he began to mend, and is now at this present writing, "earning his bread by the sweat of his brow."

The rationale of this case is simple. The exposure to cold and moisture in undue degree, converted what would have been, without such exposure, a mild paroxysm of simple intermittent into a malignant congestive chill, oppressing and depressing all the vital powers so as to prevent proper reaction, thus giving a fair representation of the congestive form of Bilious fever. If it were not so, I am at a loss to comprehend what that type of fever is, and must come down from the witness' stand as never having seen a genuine case of it. One more prominent symptom remains to be noticed. While a jaundiced hue often follows an attack of bilious fever, another colour is its most frequent concomitant. There is in the worst types of it, a peculiar pallid anemic hue. This hue can be seen in those cases which have not fully recovered from attacks of intermittent fever, or where, as is often the case, a severe attack of bilious remittent fever has been succeeded by attacks of irregular intermittent fever prolonged late in the fall or even after a frost.

In my clinical lectures at the Savannah Hospital, I have frequently diagnosticated malarial fever subjects from merely seeing them, before, I had asked a single question of the patient. In enumerating the peculiar signs of Yellow fever, I did not speak of the yellow color of the skin because I wished to reserve that point up to this period. Now, as a *general rule*, fatal cases of that disease presented that discoloration ; and an unfavorable prognosis was almost always to be formed when the skin, began to assume that color ; yet in the commencement of the epidemic during our intensely hot and dry weather, when the cases were more acute and terminated more rapidly, I saw many dead bodies whose skin could not have afforded any index to the disease of which they had died, although black vomit had been freely thrown up before death. Of the cases which did recover, although many had been very severe, very few presented any morbid discoloration of the skin, and it was a subject of frequent remark by those who returned to us after the pestilence had left us, that they were astonished to see the survivors looking so well and free from any marks of previous diseases. A gentleman, his wife and child, had all had very severe attacks of the fever in September 1854. He visited the North late in October for a change. He has often told me that persons there would hardly believe that they had just come from what was then an infected city, and that they had been sufferers from the scourge, so little did they bear any traces of it with them. Let me then sum up what are the prominent symptoms during life of each disease, before I go into the signs presented after death. I speak of the average of symptoms without noticing the varieties which occur in this, as in every other disease. In Yellow fever, the access of the disease is generally sudden ; a person may be about in the morning and quite ill in the evening, or may be well in the evening and attending to business and be prostrated in the morning following. The sympathetic pains are much greater ; the pain is over frontal region, over sacrum and down the thighs ; the skin is hot and dry, and does not pour out perspiration as in Bilious fever ; the pulse, never mind how high the febrile symptoms, seldom ranges over a hundred ; the tongue is not coated, on the contrary offers no index of the state of the stomach. The paroxysm of fever subsides without any critical evacuation and a state of calm succeeds which lasts from a few hours to forty-eight hours. The pulse at this stage generally falls as low as fifty or sixty. In bad cases, the stomach invariably shows great tenderness upon pressure, or there is an uneasy sensation in the epigastrium, and an intolerance of food. With this, there is also a remarkable prostration of strength. Many cases seem to be threatened with death from sheer exhaustion; nor is this at all dependent on any previous evacuations from the system, nor is it always in a direct ratio to the severity of the febrile paroxysm, for it would occur where there had been no evacuation, and would follow a very slight paroxysm. If the case continued to grow worse, the retching is followed by the vomiting of the black vomit, the occurrence of which at the season of the year when alone Yellow fever prevails in this climate, leaves no doubt as to the nature of the disease or the fate of the case; or hemorrhage would occur from the mouth, the lips, the tongue, the gums, a scorbutic oozing. In an epidemic of Bilious fever, many of the cases have their periodicity so well marked that no one could doubt as to their true nature. Other cases have their remissions more obscurely marked, and without close watching, would seem to be continued fever, but a close observation will generally detect marked remissions, and decided exacerbations. The remissions almost always occur in the forenoon, the exacerbation in the latter part of the day and at night. Bilious fever seldom attains its height at one bound as does Yellow fever. Questioning will frequently reveal the fact that there has been a distinct intermission between the first and second paroxysm. The pulse is most certainly more accelerated in Bilious fever, reaching often into a paroxysm up to 120 to 140. It will also vary many beats in the course of the day. A paroxysm seldom lasts longer than twenty-four hours when it either terminates, or there is a marked remission accompanied by sweats more or less profuse, and a sensible abatement of all the febrile symptoms. After this fever again rises, again runs the same round. If the case is to terminate favorably, the paroxysms become lighter and lighter, the remissions more marked; very frequently they may be considered perfect intermissions, and you will see that the great peculiarity of Bilious fever is its *periodicity*. In the paroxysms there are headache, and backache, and pain the sciatic nerve, but they are not so marked, as a general rule; not of such a neuralgic character as is often seen in Yellow fever. There are nausea and vomiting, but bile continues to be thrown up to the last stage of Bilious fever, should it be a case marked by great irritability of the stomach. Now when a person is attacked by Yellow fever, of course there is some bile in the system, and it may sometimes be thrown up at the very commencement of the attack, but certainly it is never seen in the advanced stages of the disease.

In October 1842, I published the article on Black Vomit in the American Journal of the Medical Sciences alluded to before. My experience was then limited, but I adhere without qualification to the opinion then announced by me, viz: "Perhaps there may be bile in the incipiency of the attack, before a physician is called, but in every case that has ever come under my notice, that has terminated in black vomit, *the absence of bile in the excretions has been the distinctive characteristic of the disease.*" The head is decidedly more affected in Bilious fever, than in Yellow fever. It is a common thing for patients to remain in their senses long after they have reached the stage of black vomit. Just before death, the brain gives way as the other organs do. But in a bad case of Bilious fever there is almost always oppression of the brain, and cases lie in a stupor for two or three days before death. I think the anatomical lesions discovered in Bilious fever after death, which, I will detail further on, sufficiently account for this.

I think I have furnished sufficient points of contrast for the symptoms during life. Let us follow out the diseases, and see what anatomical lesions are left on the dead body by them.

In Bilious fever we find marks of disease on the mucous coat of the stomach, the upper part of the duodenum and the liver. These I may state as invariable. In a large number of cases, and particularly in the worst types of Bilious fever, there are traces of the disease on and under the meninges of the brain.

In Yellow fever we find the same organs affected, except that, as the brain is involved during life in but comparatively few cases, it does not exhibit the same uniform alteration as do the stomach and liver. Now here is a point of relation, and to what does it amount?—to no more than does the relation of scarlet fever to measles, in each of which the skin and air passages are affected. In Bilious fever we find what I consider undoubted marks of an inflamed stomach.

The mucous membrane is often red and injected, either punctated or arborescent, it is often softened, so as it can be easily scraped off with the handle of the scalpel; it is very often of a slate color in protracted cases, but *invariably* traces of bile can be detected in the stomach or in the intestines. In Yellow fever, we also find the mucous membrane injected, but certainly much more generally and much more intensely than in Bilious fever. You do not always find black vomit in the stomach, because it may have been ejected just before death, but most generally you will. I have opened, in my day, several subjects dead from Yellow fever, in whose stomach black vomit was found, although not a particle had been thrown up. The patient, from Augusta, who entered the hospital in 1839, alluded to before, was one of these cases. Without a post-mortem examination, it might have remained in doubt as to what his fever was. Another case, amongst the very last of 1856, was examined after death by Dr. J. B Read and myself, and black vomit was found in the stomach, although none had been ejected during life.

Black vomit is however generally found in the stomach; but it is found free in almost all instances, lying on the surface of the mucous membrane; but there are cases in which the peculiar flocculi of black vomit can be detected in the very mouths of the patulous vessels of the mucous membrane; and in some cases, I have seen a dark black arborescent injection running under or in the mucous membrane, exactly like the red arborescent injection so frequently met with. Now black vomit is a hemorrhage. I expressed in 1842, (loc. cit.) my belief, that it was blood altered; in 1852, I detected blood corpuscles existing in it; I exhibited them under the microscope to Dr. Wragg, Dr. West, Dr. Read, Dr. Bulloch, and the late Dr. Ladd. Here is proof positive that the mucous membrane is the seat of a peculiar hemorrhage. Dr. Copland has some grounds in wishing to designate Yellow fever as the hæmagastric pestilence. Whatever may be the real poison, it undoubtedly has a peculiar tendency to produce hemorrhage from the stomach; but there is a great deal of acid in the stomach, and it produces a peculiar effect on the blood, coagulating it into the flocculi of black vomit. The ejecta in Yellow fever, tested by litmus paper, always show strong acidity; it is the acid which turns the blood, and prevents the hemorrhage from being a mere hematemesis. Occasionally the hemorrhagic tendency of the disease is shown by its action on the bowels, and blood is passed downwards. Such cases, as far as my experience goes, are always fatal, are genuine Yellow fever; but must be distinguished from those in which the black vomit is passed per anum; in the latter case, recovery is more apt to follow than when the unaltered blood is passed.

Now, if Yellow fever were but the highest grade of Bilious fever, we ought every season to meet with occasional cases in which black vomit would be found in the stomach after death, even if it were not ejected during life. Such cases have never occurred in experience. But it is when we examine the liver that we find unmistakable evidences of the peculiar nature of Yellow fever. In Bilious fever we find the liver of various shades, dark brown. umber, bronze, but always gorged with blood. In Yellow fever it is always altered in color, being pale and destitute of blood. The best color to which I can compare it is boxwood. Some boxwood is of a dirty yellow, some of a brighter yellow; so of the liver, some are of a light pale boxwood, almost a dirty ash white, some of a more pronounced yellow color. In a Bilious fever liver, by pressing a piece of white paper on the cut acini, you will stain it yellow, showing the secretion of bile still having existed up to the time of death; but this cannot be done with a Yellow fever liver. It sometimes contains a thin bloody serum, most generally it is almost dry. In 1827, Dr. Warning pointed out to me this state of the liver as the exact state presented in our fatal epidemic of 1820. In every case that I have had the opportunity of examining from that time to the present, I have found the identical appearances. I examined cases at all times of our epidemic of 1854, and I found no variation of any account. I consider this conclusive proof of the identity of the disease, from 1820 to 1854.

One case early in the season, presented a mottled liver, that is, there were spots in it which had undergone the peculiar change incident to Yellow fever, and there were other spots in which the liver presented the natural Spanish brown color. I attribute this to the patient having died before the change in the circulating fluids had been sufficiently great to effect the alteration of the entire parenchyma. In some cases of Yellow fever, I have seen the gall bladder contain only a dirty, thick, viscid bile. In Bilious fever, it is always filed with bile.

In Yellow fever, the absence of bile is not confined to the stomach: you may search from the cardiac orifice to the anus without finding a trace of it; often have I done so, and never have I succeeded. I do not say, that a person who has the opportunity of examining *every* fatal case which may occur in a large hospital may not succeed better, but when a man is harrassed with constant demands on his time, as a physican in full private practice will be in great epidemics, he cannot examine every case. It was my object in 1854, to procure a record of the post-mortem appearances during the various periods of the epidemic, and I examined cases which died in August, September, October, and November, and I found a great uniformity in all of them—the discolored liver and the total absence of biliary secretion in the primæ viæ.

I was the attending physician of the Savannah Hospital for every summer, from that of 1835 to that of 1849, inclusive. I rarely allowed a fatal case of Bilious fever to escape without an autopsy. I state most distinctly, that in every case I found an abundance of bile in the intestines, if I did not find it in the stomach.

My examinations of the head in Yellow fever have been very few. In the great majority of cases, the cerebral symptoms did not induce me to do so. I have seen cases in which the head was involved from the commencement, and, doubtless, those cases would have furnished evidences of cerebral engorgement; but, as a general rule, the local manifestations of Yellow fever are in the stomach and liver. The existence of long continued stupor in Bilious fever made me examine the brain very frequently; indeed, in the great majority of cases, and as I stated before, enough was found to account satisfactorily for the stupor;—serum was generally effused under the coverings of the brain, in the ventricles, and under the arachnoid, and the latter membrane was frequently opalescent. I have always considered the brain as a special organ for the local manifestation of Bilious fever, both from the decided symptoms presented during life, and from the post-mortem appearances.

I present for the inspection of the Society, drawings in oil colors taken from nature, of the appearances of the liver in the two diseases. An inspection will be better than any description. The Society cannot fail to see, at a glance, the vast difference from the brown and bronze of the four copies of the Bilious fever livers, and the light yellow boxwood-color of the Yellow fever liver. It has been my object to sketch the prominent characteristics of the two diseases, presented during life and after death. If they be so widely and so uniformly different, how can we class them as the same disease, modified only by intensity?

The second opinion entertained, that Yellow fever is a disease, *sui generis*, special, distinct, has been sufficiently discussed in the consideration devoted to the opinion, whether it is only a higher grade of Bilious fever. It will be seen that I entertain this belief.

The question of its origin and propagation wonld, itself, afford scope for an essay; and mine has already occupied so much time, that I could not go into it now. I can bear my decided testimony that in no instance has there ever been the shadow of the shade of proof, that it ever was imported into Savannah from abroad. On the contrary, the proof is positive that its first victims had had no communication, direct or indirect, with any source of infection. Moreover, when the British steamer Conway, which ran to the West Indies, touched at this port, I attended two cases of Yellow fever from her, both of which died in the city, and yet no disease was propagated from them. In March, 1841, (as will be seen by my article quoted before,) I brought a case from a ship from Demerara, and placed it in the hospital where the patient died. It is said that it can be propagated from abroad in a city, although most give up the point as to its contagion in the country. The whole experience of 1820 and 1854, when our citizens fled by hundreds into the country, and into neighboring villages, towns, and cities, does not afford a single instance where the disease was spread by the fugitives. If then, it is not propagated into the country, and into other cities by land routes, why is it supposed to be so fatal when it comes by sea? If one case can originate in a place, why not ten or twenty? Case upon case occurred in 1854, in which the patients had not been near a deceased subject. Isolation was no protection. The poison, whatever it may be, spread like a pall over the whole city, and covered in its embrace all who staid, or entered its precincts; but a quarter of a mile beyond its limits the poison became innocuous. Such is fact. Let those who appeal to fancy, disprove it, or theorise upon it.

Again: facts prove that Yellow fever is a city disease. Exposure to swamp malaria, staying on a rice plantation in the summer, and in the fall before a frost, will produce a malignant and most fatal Congestive-bilious fever; but *never*, no *never* Yellow fever. Such cases of Bilious fever, as I stated before, I meet with every year; but, thank God, very seldom have I encountered cases of Yellow fever.

Yellow fever, in this locality, has this in common with Bilious fever, it never prevails except in the summer and fall months, and is most effectually cut short by a frost. As a general epidemic, it ceased to prevail in Savannah about the second week in October in 1854; yet the poison continued in the atmosphere until a frost, and attacked those strangers who imprudently returned into the city. The last resident whom I attended was attacked on the 25th of October. The cases which occurred afterwards were, without exception, strangers and unacclimated.

Since my connexion with the Savannah Medical College, I again attend the hospital, and it was there, and amongst seamen that I met with my last cases. They lay promiscuously amongst patients with other diseases, but in no single instance did any body catch the disease. I stated towards the close of that ever memorable season, that I would expect to meet with Yellow fever for a fortnight after a frost. I had taken up the belief that ten days, or a fortnight, was the period of incubation of the poison. My last case died at the hospital on the 27th of November; frost had occurred on the 13th of that month. The unfortunate subject had reached our city before the mighty destroyer of the poison had withered and destroyed its noxious powers.

Such, gentlemen, is my *experience* of the relation of Yellow and Bilious fever.—*Southern Medical & Surgical Journal.*

SAVANNAH, April, 1856.

